# Avian Paramyxovirus 4 Antitumor Activity Leads to Complete Remissions and Long-term Protective Memory in Preclinical Melanoma and Colon Carcinoma Models

**DOI:** 10.1158/2767-9764.CRC-22-0025

**Published:** 2022-07-07

**Authors:** Aryana Javaheri, Yonina Bykov, Ignacio Mena, Adolfo García-Sastre, Sara Cuadrado-Castano

**Affiliations:** 1 Department of Microbiology, Icahn School of Medicine at Mount Sinai, New York.; 2 Global Health and Emerging Pathogens Institute, Icahn School of Medicine at Mount Sinai, New York.; 3 Department of Medicine, Icahn School of Medicine at Mount Sinai, New York.; 4 The Tisch Cancer Institute, Icahn School of Medicine at Mount Sinai, New York.; 5 Department of Pathology, Molecular and Cell-Based Medicine, Icahn School of Medicine at Mount Sinai, New York.

## Abstract

Avulaviruses represent a diverse subfamily of nonsegmented negative-strand RNA viruses infecting avian species worldwide. To date, 22 different serotypes have been identified in a variety of avian hosts, including wild and domestic birds. APMV-1 (Avian Paramyxovirus 1), also known as Newcastle disease virus (NDV), is the only avulavirus that has been extensively characterized because of its relevance for the poultry industry and, more recently, its inherent oncolytic activity and potential as a cancer therapeutic. An array of both naturally occurring and recombinant APMV-1 strains has been tested in different preclinical models and clinical trials, highlighting NDV as a promising viral agent for human cancer therapy. To date, the oncolytic potential of other closely related avulaviruses remains unknown. Here, we have examined the *in vivo* antitumor capability of prototype strains of APMV serotypes -2, -3, -4, -6, -7, -8, and -9 in syngeneic murine colon carcinoma and melanoma tumor models. Our studies have identified APMV-4 Duck/Hong Kong/D3/1975 virus as a novel oncolytic agent with greater therapeutic potential than one of the NDV clinical candidate strains, La Sota. Intratumoral administration of the naturally occurring APMV-4 virus significantly extends survival, promotes complete remission, and confers protection against rechallenge in both murine colon carcinoma and melanoma tumor models. Furthermore, we have designed a plasmid rescue strategy that allows us to develop recombinant APMV-4–based viruses. The infectious clone rAPMV-4 preserves the extraordinary antitumor capacity of its natural counterpart, paving the way to a promising next generation of viral therapeutics.

**Significance:**

Discovery of the oncolytic properties of APMV-4 Duck/Hong Kong/D3/1975: a novel cancer therapeutic with natural capacity to exert complete remission and long-term antitumor protection in syngeneic mouse cancer models.

## Introduction

**FIGURE 1:**
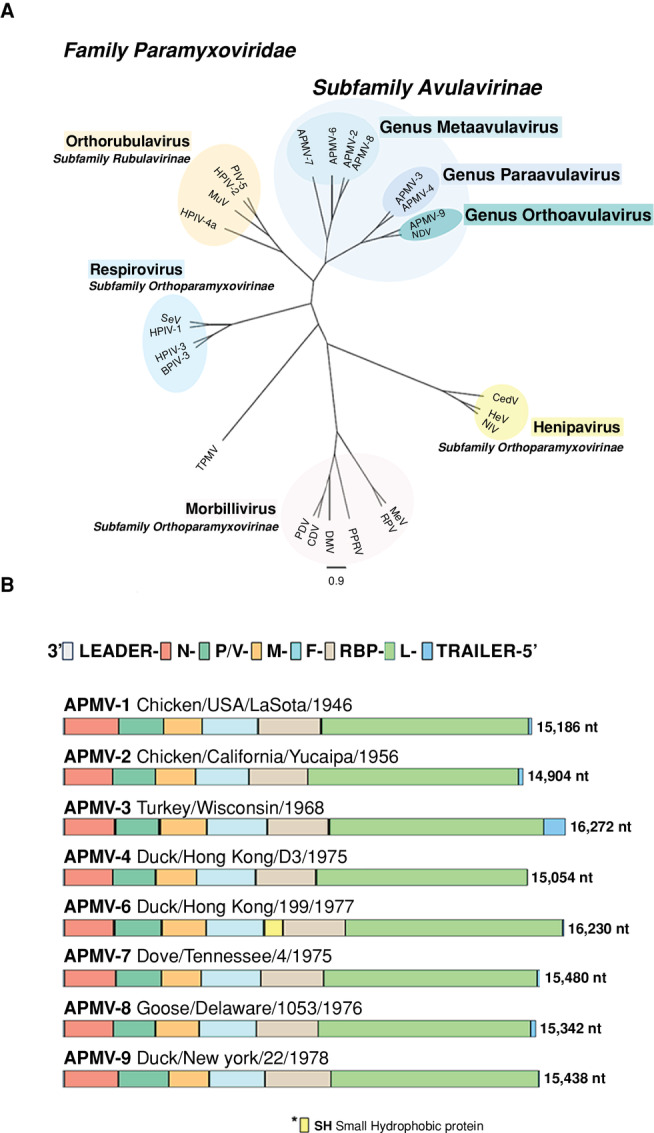
Selection of APMV viruses. **A,** Phylogenetic tree of the attachment protein of representative paramyxoviruses. Maximum likelihood phylogenetic tree was created using MEGA-using amino acid sequence of the attachment proteins from the reference viruses of important human and animal paramyxoviruses, as well as the 8 avian avulaviruses used in this study. **CedV:** Cedar virus. **HeV:** Hendra virus. **NIV**: Nipah virus. **MeV**: Measles virus. **RPV**: Rinderpest virus. **PPRV**: Peste des petits ruminants’ virus. **DMV**: Dolphin morbillivirus. **CDV**: Canine distemper virus. **PDV**: Phocine distemper virus. **TPMV**: Tupaia paramyxovirus. **BPIV-3**: Bovine parainfluenza virus 3. **HPIV-3**: Human respirovirus 3. **HPIV-1**: Human parainfluenza virus 1. **SeV**: Sendai virus. **HPIV-4a**: Human parainfluenza virus 4a. **MuV**: Mumps orthorubulavirus. **HPIV-2**: Human rubulavirus 2. **PIV-5**: Parainfluenza virus 5. **APMV-7**: Avian paramyxovirus 7. **APMV-6**: Avian paramyxovirus 6. **APMV-2**: Avian paramyxovirus 2. **APMV-8**: Avian paramyxovirus 8. **APMV-3**: Avian paramyxovirus 3. **APMV-4**: Avian paramyxovirus 4. **APMV-9**: Avian paramyxovirus 9. **NDV**: Newcastle Disease Virus LaSota strain. **B,** Schematic representation of genome organization of selected APMV viruses isolates in studies**.** Size of the viral genes is shown to scale.

The *Paramyxoviridae* family of viruses includes numerous important pathogens that impact both humans (mumps, measles) and animals (Sendai, Hendra, and Nipah viruses). Paramyxoviruses are enveloped pleomorphic viruses containing a nonsegmented, negative-sense, single-stranded RNA genome with a viral life cycle limited to the cytoplasm of the host cell. All paramyxoviruses isolated from avian species are classified into the subfamily *Avulavirinae*. The most recent taxonomic revision of the group differentiated 22 avian paramyxovirus (APMV) species distributed into three different genera (Fig. 1A): *Metaavulavirus, Orthoavulavirus, and Paraavulavirus* (1). APMVs have been isolated from a wide range of domestic and wild birds. Clinically, infection presentation varies in a strain-specific and host-dependent manner, from asymptomatic to high rates of morbidity and mortality (2). With a size range of 14,900–17,000 nucleotides, the genome of APMVs encodes six structural proteins involved in the viral replication cycle (Fig. 1B): the nucleoprotein (N), phosphoprotein (P), and large polymerase protein (L) are, in association with the viral RNA, the components of the ribonucleotide protein complex (RNP). The RNP exerts dual functionality, acting as both the (i) nucleocapsid and (ii) replication machinery of the virus. The matrix protein (M) assembles between the viral envelope and the nucleocapsid, participating actively during virus assembly and budding. The receptor-binding protein (RBP) HN and fusion (F) glycoproteins drive the initiation of the infection: the RBP HN targets neuraminic acid–containing proteins as receptors at the host cell surface, subsequently triggering the activation of the F protein, promoting fusion of the viral and cell membranes, which allows the entry of the RNPs to the cytoplasm. Genome replication is carried out within the cytoplasm by the viral RNA-dependent RNA polymerase and does not involve any DNA-intermediate stage. After each round of replication, the newly generated viruses are released from the host cell by budding followed by detachment mediated by RBP HN neuraminidase activity (3).

Avian paramyxovirus 1 (APMV-1), commonly known as Newcastle disease virus (NDV), is the most extensively characterized member of the avulaviruses due to the high mortality rate and economic loss caused by virulent strains in the poultry industry (4). Despite the devastating impact of highly pathogenic strains, NDV can be controlled by the prophylactic administration of live-attenuated and/or killed virus vaccines (5). NDV strains are classified as either velogenic (highly virulent), mesogenic (intermediate virulence), or lentogenic (low-virulence or avirulent), in accordance with the severity of the clinical signs displayed by affected chickens (6, 7). Regardless of its prevalence and worldwide distribution, NDV strains do not represent a human threat. Occasional human infections are restricted to direct contact with sick birds and resolved with mild flu-like symptoms or conjunctivitis (8). Reported NDV infections in mammals have demonstrated that these avian viruses are neither capable of establishing persistent infection nor counteracting the restriction in host tropism in mammalian cells (9, 10). Furthermore, different strains of NDV have been shown to act as strong stimulators of humoral and cellular immune responses at both the local and systemic levels (11–13). Because of the safety and immunostimulatory properties of NDV in mammals, and because the development of a reverse genetics system (14, 15) that allows us to manipulate the genome of negative-strand RNA viruses, several NDV strains have been used as vaccine vectors in poultry, mammals, and humans to express antigens of different pathogens (16–18).

Over the past three decades, there has been increased interest in the use of NDV as an antineoplastic agent. The inherent antitumor capacity of NDV combines the two major characteristics that define an oncolytic virus (OV): induction of tumor cell death (19), accompanied by the elicitation of antitumor immunity and long-term protection (20, 21). From initial reports on its antitumoral potential in the mid-19th century until now, various NDV strains have been utilized in animal models and/or patients with cancer by different routes (intratumoral, locoregional, or systemic), using a multitude of therapeutic approaches, such as viral oncolysates, live cell tumor vaccines, or DC vaccines pulsed with viral oncolysate (22). Nowadays, many research groups, including ours, work toward the development of more efficient NDV-based antitumor strategies (23–27).

In contrast to what is known about NDV strains, there is limited information on the biology of other avian avulavirus isolates, and there have been no previous studies assessing the oncolytic potential of other closely related APMVs. Here we probed the *in vivo* oncoytic capacity of other members of the *Avulavirinae* subfamily. Preclinical syngeneic murine melanoma and colon carcinoma tumor models were challenged intratumorally with prototype viruses from APMV-2, -3, -4, -6, -7, -8, and -9 species so as to compare their survival outcomes with those induced by the clinical candidate NDV LaSota L289A virus (LS-L289A).

## Material and Methods

### Cell Lines, Antibodies, and Other Reagents

Murine cancer cell lines B16-F10 (mouse skin melanoma cells; ATCC, catalog no. CRL-6475) and CT26.WT (mouse colon carcinoma cells; ATCC, catalog no. CRL-2638) were maintained in RPMI medium supplemented with 10% FBS and 2% penicillin and streptomycin. Human melanoma SK-MEL-2 (ATCC, catalog no. HTB-68), colon carcinoma RKO-E6 (ATCC, catalog no. CRL-2578), and human normal colon fibroblast CDD-18Co cells (ATCC, catalog no. CRL-1459) were propagated using supplemented ATCC-formulated Eagle Minimum Essential Medium. African green monkey kidney epithelial Vero cells (ATCC, catalog no. CCL-81), human normal skin fibroblast HFF-1 cells (ATCC, catalog no. SCRC-1041), and BSR-T7 cells (28) were maintained on supplemented DMEM. Master cancer cells banks were created after purchase and early-passage cells were thawed in every experimental step. Once in culture, cells were maintained no longer than 8 weeks to guarantee genotypic stability and were monitored routinely by microscopy. *Mycoplasma* testing was performed once a month using MycoStript-*Mycoplasma* Detection Kit from InvivoGen (catalog no. rep-mys-50). Required IMPACT validation test for cancer cells involved in our *in vivo* experiments was performed by the Center for Comparative Medicine and Surgery at Icahn School of Medicine at Mt. Sinai (Mount Sinai Hospital, New York, NY). Reduced serum media Opti-MEM (Gibco) was used for *in vitro* viral infection medium. Rabbit polyclonal serum to NDV was described previously (10). APMV serotype-specific antiserums APMV-2 (471-ADV), APMV-3 (473-ADV), type-4 475-ADV, type-6 479-ADV, type-7 481-ADV, type-8 483-ADV, and type-9 485-ADV, 2017) were purchased from the National Veterinary Services Laboratories, United States Department of Agriculture (USDA, Ames, IA). Goat anti-chicken, Alexa-conjugated secondary antibody (Alexa-568, A-11041) was sourced from Thermo Fisher Scientific. Hoechst 33258 nuclear staining reagent was purchased from Invitrogen (Molecular Probes).

### Viruses

Modified NDV LaSota-L289A has been described previously (29). APMV viruses isolates were obtained from National Veterinary Services Laboratories, USDA (Ames, IA): APMV-2 Chicken/California/Yucaipa/1956 (171ADV9701), APMV-3 Turkey/Wisconsin/1968 (173ADV9701), APMV-4 Duck/Hong Kong/D3/1975 (175ADV0601), APMV-6 Duck/HongKong/199/1977 (176ADV8101), APMV-7 Dove/Tennessee/4/1975 (181ADV8101), APMV-8 Goose/Delaware/1053/1976 (October 27, 1986), and APMV-9 Duck/New York/22/1978 (185ADV 0301). Viral stocks were propagated in 9-day-old embryonated chicken eggs and clear purified from the allantoic fluid by discontinuous sucrose density gradient ultracentrifugation for resuspension and storage in PBS. Viral titers were calculated by indirect immunofluorescence on Vero cells.

### Phylogenetic Tree of the Attachment Protein of Representative Paramyxoviruses

The amino acid sequence of the attachment protein (RBP, H or G) from the reference viruses of important human and animal paramyxoviruses, as well as those of the 8 APMVs used in this study, were downloaded from GenBank. The sequences were aligned and a maximum likelihood phylogenetic tree was created using MEGA-X v10.0.5. The tree was visualized with FigTree v1.4.3. CedV: Cedar virus (NC_025351.1). HeV: Hendra virus (NC_001906.3). NIV: Nipah virus (NC_002728.1). MeV: Measles virus (NC_001498.1). RPV: Rinderpest virus (NC_006296.2). PPRV: Peste des petits ruminants virus (NC_006383.2). DMV: Dolphin morbillivirus (NC_005283.1). CDV: Canine distemper virus (NC_001921.1). PDV: Phocine distemper virus (NC_028249.1). TPMV: Tupaia paramyxovirus (NC_002199.1). BPIV-3: Bovine parainfluenza virus 3 (NC_002161.1). HPIV-3: Human respirovirus 3 (AB012132.1). HPIV-1: Human parainfluenza virus 1 (NC_003461.1). SeV: Sendai virus (NC_001552.1). HPIV-4a: Human parainfluenza virus 4a (NC_021928.1). MuV: Mumps orthorubulavirus (NC_002200.1). HPIV-2: Human rubulavirus 2 (NC_003443.1). PIV-5: Parainfluenza virus 5 (NC_006430.1). Sequence references for APMVs have been included in Table 1.

**TABLE 1:** Selected APMVs included in the studies

GENUS	SPIECES	ISOLATE	NATURAL HOST	PATHOGENICITY IN CHICKENS	SEQUENCE
**ORTHO AVULAVIRUS**	**APMV-1**	Chicken/USA/LaSota/1946	Chicken	Avirulent^a^; MDT: 110 h; ICP: 0 (31)	JF950510.1
	**APMV-9**	Duck/New York/22/1978	Domestic and feral duck	Avirulent^a^; MDT > 120 h; ICP: 0 (34)	NC_025390.1
**METAAVULAVIRUS**	**APMV-2**	Chicken/California/Yucaipa/1956	Chicken and turkey	Avirulent^a^; MDT > 168 h; ICP: 0 (35)	EU338414.1
	**APMV-6**	Duck/Hong Kong/199/1977	Duck, geese, turkey	Avirulent^a^; MDT > 168 h; ICP: 0 (37)	EU622637.2
	**APMV-7**	Dove/Tennessee/4/1975	Hunter-killed dove, turkey and ostrich	Avirulent^a^; MDT > 144 h; ICP: 0 (36)	FJ231524.1
	**APMV-8**	Goose/Delaware/1053/1976	Feral Canadian goose and pintail	Avirulent^a^; MDT > 144 h; ICP:0 (33)	FJ619036.1
**PARA AVULAVIRUS**	**APMV-3**	Turkey/Wisconsin/1968	Turkey and parakeet	Avirulent^a^; MDT > 168 h; ICP: 0 (31)	EU782025.1
	**APMV-4**	Duck/Hong Kong/D3/1975	Wild and mallard duck, chicken, geese	Avirulent^a^; MDT > 144 h; ICP: 0 (32)	FJ177514.1

^a^Growth in 9-day-old embryonated chicken eggs.

**Abbreviations:** MDT, mean embryo death time is the mean time in hours for the minimal lethal dose to kill inoculated embryos. Virulent, 60 hours; intermediate 60–90 hours; avirulent >90 hours. ICP, intracerebral pathogenicity index: evaluation of disease and death following intracerebral inoculation in 1-day-old SPF chicks. Virulent 1,5–2; intermediate 0.7–1.5; avirulent strains 0.7–0.0.

### Amplification and Cloning of a Full-length cDNA of the APMV-4 Genome

Viral RNA was purified from an egg-grown viral stock, following manufacturer instructions from the E.Z.N.A. viral RNA kit (Omega). Next, the viral RNA was used as a template to amplify overlapping fragments of the genome's cDNA using RT-PCR kit SuperScript IV One-Step RT-PCR System (Invitrogen). PCR primers (Supplementary Table S1) were designed to allow cloning by InFusion (Clontech) and to introduce unique restriction sites at nonconserved parts of each intergenic region. Next, the RT-PCR products were cloned into plasmid pUC-18 to obtain intermediate plasmids pUC-APMV4-1, pUC-APMV4-2, and pUC-APMV4-3. Using restriction digestion and ligation, we first combined fragments 1 and 2 into pUC-APMV4-1+2, and then added fragment 3 to obtain pUC-APMV4-1+2+3, which contains the full-length copy of our viral cDNA. Finally, the complete genome sequence was transferred to the rescue plasmid (pAPMV4) under the control of the T7 promoter and a self-cleaving hammerhead ribozyme at 5′, and a self-cleaving hepatitis delta ribozyme and T7 terminator at 3′.

### Cloning of the Helper Plasmids pTM1-N, pTM1-P, and pTM1-L

The open reading frames of the viral genes N, P, and L were amplified from the intermediate plasmids pUC-APMV4-1 (N and P genes), and pUC-APMV4-3 (L gene) and cloned by InFusion into the expression plasmid pTM-1 under the control of the T7 promoter.

### Rescue of Infectious Recombinant APMV4 by Plasmid Transfection

To rescue the recombinant APMV4, we followed the protocol used in our laboratory to rescue NDV with the APMV-4–specific plasmids described above. In brief, BSR-T7 cells growing in a 6-well plate were infected with recombinant vaccinia virus MVA-T7 for 1 hour at 37°C, and then transfected with helper plasmids pTM1-N (0.5 μg), pTM1-P (0.25 μg) and pTM1-L (0.25 μg), and rescue plasmid pAPMV4 (1 μg). This transfection was incubated at 37°C for 24 hours, following which cells and supernatant were inoculated into 8-day-old embryonated chicken eggs to amplify the rescued virus.

### Fluorescence Microscopy

For indirect immunofluorescence staining, the cells were infected for 1 hour at the indicated multiplicity of infection (MOI) in Opti-MEM, after which the inoculum was supplemented with cell-specific complete medium. Cell fixation was performed using 2.5% paraformaldehyde for 15 minutes. Cell-membrane permeabilization was carried out using 0.2% Triton-PBS for 10 minutes and blocked in PBS 1% BSA for 1 hour. The samples were incubated with specific primary antibodies at 1:400 dilution for 1 hour at room temperature. Secondary antibodies (goat anti-chicken Alexa Fluor 568, goat anti-rabbit Alexa Fluor 488; purchased from Invitrogen) were used at a 1:800 dilution for 45 minutes prior to imaging using an EVOS FL cell imagine system (Thermo Fisher Scientific).

### *In Vitro* Cell Viability MTT Assay

Cancer cells were cultured at a confluence of 80% in 24-well plates and infected with our APMV viruses in Opti-MEM at the indicated MOI for 1 hour; cells were then supplemented with complete media. After 24 hours of incubation, the infection media was removed and cells were incubated for 1 hour and 15 minutes with 300 μL of 2.5 mg/mL MTT at 37°C, under light-restricted conditions. Resulting formazan crystals were dissolved with 700 μL of Isopropanol through manual disturbance and a 10-minute, light-restricted incubation on a shaker. The absorbance of each sample was recorded at 570 nm using a BioTek plate reader.

### Multistep Replication Kinetics

Cancer cells monolayers in 6-well plates were infected with the specified viruses at a MOI of 0.1 PFU/cell in OptiMEM-I. After 1 hour, the infection media was removed and cells were subsequently incubated with 3 mL of cell specific medium supplemented with 0.3% BSA and 1 μg/mL of TPCK-treated trypsin, to allow for production of fusion-competent viruses. Supernatants were collected at 24, 48, 72, and 96 hours postinfection and titrated by immunofluorescence assay on Vero cells using a polyclonal antiserum specific for each virus serotype.

### Transcription Analysis by qRT-PCR

Cancer cells were mock treated or infected with specified virus at a MOI of 1 PFU/cell in 250 μL of OptiMEM-I. After allowing virus adsorption for 1 hour, the cells were incubated with an additional 750 μL of supplemented media. Total RNA was isolated using a Qiagen RNeasy Minikit (catalog no.74106, Qiagen) at the indicated time postinfection. cDNA synthesis was performed using the Maxima First Strand cDNA Synthesis Kit for qRT-PCR (catalog no. K1671, Thermo Fisher Scientific). Mean *n*-fold expression levels of cDNA from three individual biological samples were normalized to 18S rRNA levels and calibrated to mock-treated samples according to the 2^−ΔΔ^*^C^*_T_ method (30). Heatmaps were created using Morpheus, https://software.broadinstitute.org/morpheus. Human and murine primer sequences have been compiled in Supplementary Table S2.

### Animal Studies

All animal studies were performed in accordance with Institutional Animal Care and Use committee (IACUC) guidelines and have been approved by the IACUC of Icahn School of Medicine at Mount Sinai (IACUC-2014-0234). Six to 8 weeks old of age female C57BL/6J and BALB/cJ mice used in our *in vivo* studies were purchased from Jackson Laboratory. B16-F10 or CT26.WT cancer cell suspension (2.5 × 10^5^ cells in 100 μL of RPMI) was intradermally implanted into the flank of the right hind leg of each experimental animal. Tumor-bearing mice were treated by intratumoral injection of the indicated viruses or PBS with doses of 5 × 10^6^ PFU/50 μL PBS, in our *in vivo screening* studies, or 10^7^ PFU/50 μL PBS in *in vivo characterization of rAPMV-4* studies. In our *in vivo* screening studies, tumors were allowed to grow to 50 mm^3^ before treatment initiation. For *in vivo* characterization of rAPMV-4 studies, B16-F10 tumors were allowed to grow to 40 mm^3^, while CT26.WT treatment commenced at 60 mm^3^. The intratumoral injections were administered every other day for a total of four treatment doses. Tumor volume was monitored every 48 hours, and every 24 hours as tumor volumes approached the experimental endpoint (EPP) of 1,000 mm^3^. Mice were humanely euthanized on the day in which the volume exceeded the predefined endpoint or at any sign of distress, including upon tumor ulceration. Tumor measurement was determined using a digital caliper, and total volume was calculated using the formula: Tumor volume (*V*) = *L*^2^ × *W*, where *L* (tumor length) is the larger diameter and *W* (tumor width) is the smallest diameter.

#### Rechallenge Studies

A total of 5 × 10^5^ cancer cells were engrafted into the contralateral hind leg of mice that shown long lasting complete responses (CR). Age-matching naïve mice were used as control for (i) tumor development and (ii) growth. No viral therapy was used in the postchallenge stage of these studies. Tumor volume was monitored every 48 hours, and mice were humanely euthanized on the day in which the volume exceeded the predefined endpoint (EPP) of 1,000 mm^3^.

### Statistical Analysis

Data analysis was performed using GraphPad Prism 9. One-way ANOVA or two-way ANOVA were used to compare multiple groups with one or two independent variables, respectively. Results are expressed as mean value ± SEM or ± SD as indicated. Comparisons of survival curves were performed using the log-rank (Mantel–Cox) test. Survival analysis for each experimental group and study was carried out using the Kaplan–Meier method. *P* values > 0.05 were considered statistically nonsignificant (ns); *, P < 0.05; **, P < 0.01; ***, P < 0.001; ****, P < 0.0001.

### Data Availability

The data generated in this study are available within the article and its Supplementary Data.

## Results

### *In Vivo* Screening of Antitumor Capacity of Selected APMV Viruses in Murine B16-F10 Melanoma and CT26.WT Colon Carcinoma Tumor Models

**FIGURE 2:**
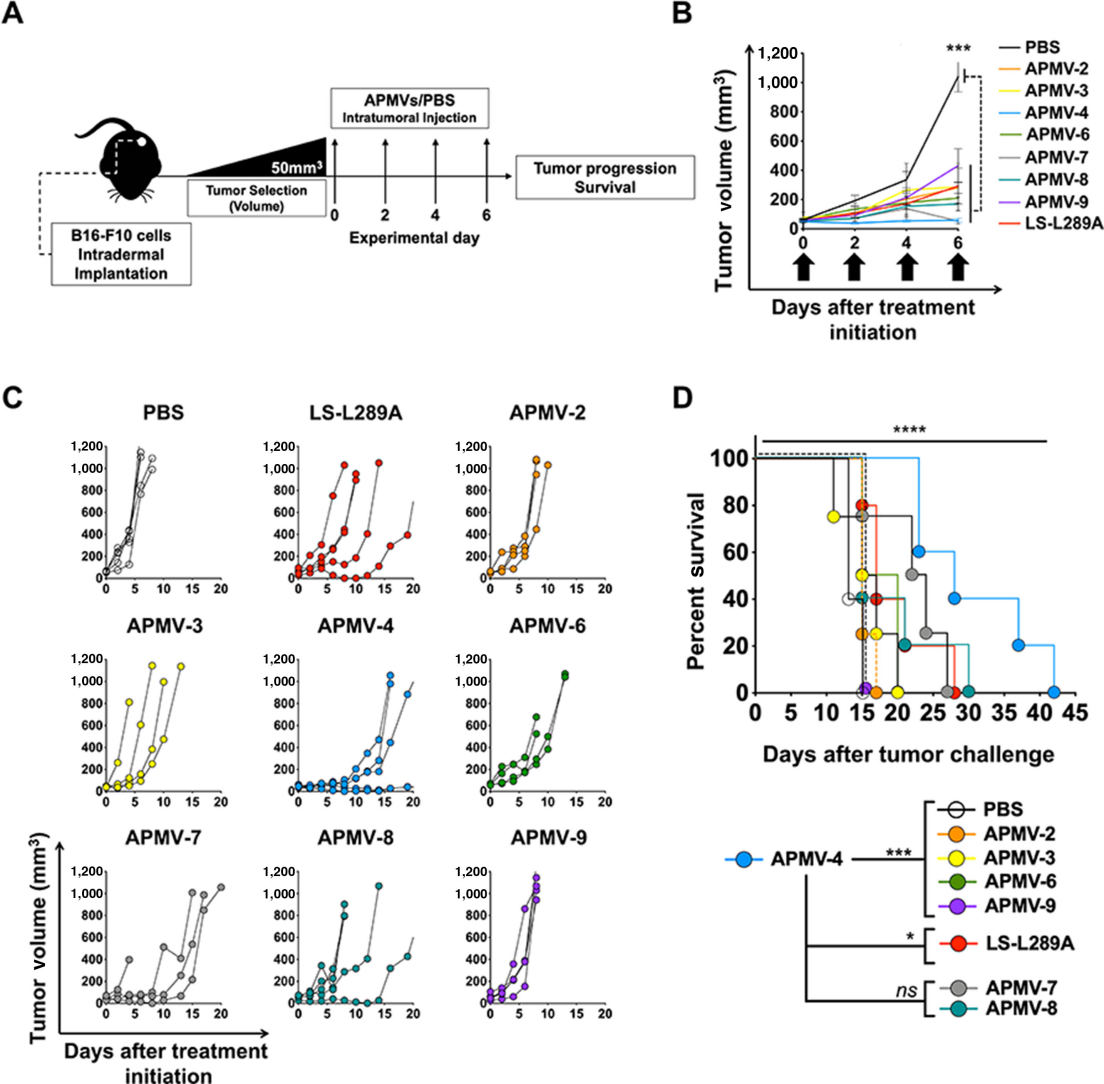
Oncolytic potential of APMV prototype viruses (I): *In vivo* screening in B16-F10 murine melanoma tumor model. **A,** Schematic representation of the study. A total of 2.5 × 10^5^ B16-F10 cells were intradermally implanted on the right hind leg. Tumor-bearing mice were treated intratumorally every other day with a total of four doses of 5 × 10^6^ PFU of LS-L289A, APMV prototypes, or PBS for control mice (days 0, 2, 4, and 6). Tumor volume was monitored every 48 hours. EEP of 1,000 mm^3^. **B,** Average of tumor volumes for each experimental group on days of therapy administration ± SEM. **C,** Individual tumor growth curves. Each point represents tumor volume per mice at the indicated timepoint. **D,** Survival analysis. *P* values obtained through log-rank (Mantel–Cox) statistical analysis. *N* = 5 animals for PBS, LS-L289A, APMV-4, -8, and -9 experimental groups. *N* = 4 animals for APMV-2, -3, -6, and -7 groups. *, *P* < 0.05; ***, *P* < 0.001; ****, *P* < 0.001; *ns*, nonsignificant.

**FIGURE 3:**
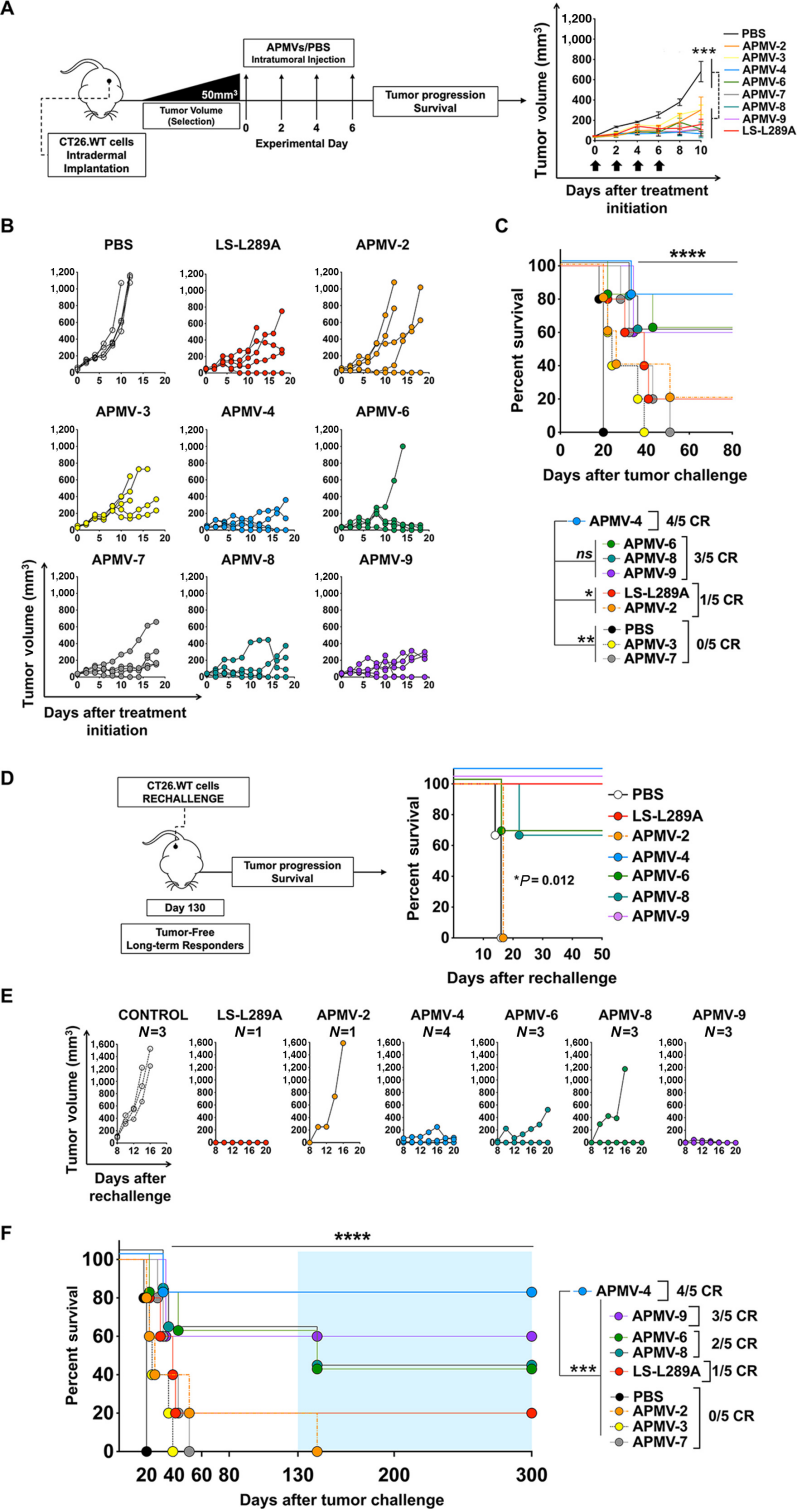
Oncolytic potential of APMV prototype viruses (II): *In vivo* screening in CT26.WT murine colon carcinoma tumor model. **A,** Schematic representation of the study. A total of 2.5 × 10^5^ CT26.WT cells were intradermally implanted on the right hind leg. Tumor-bearing mice were treated intratumorally every other day with a total of four doses of 5 × 10^6^ PFU of LS-L289A, APMV prototypes, or PBS for control mice (days 0, 2, 4, and 6). Tumor volume was monitored every 48 hours. EEP of 1,000 mm^3^. Right: average of tumor volumes for each experimental group on days of therapy administration ± SEM. **B,** Individual tumor growth curves. Each point represents tumor volume per mice at the indicated timepoint. **C,** Survival analysis at 80 days. *P* values obtained through log-rank (Mantel–Cox) statistical analysis. CR, complete response. *N* = 5 animals for each experimental group. **D–F,** Rechallenge, tumor-free long-term responders were reimplanted with 5 × 10^5^ CT26.WT cells in the contralateral-left-hind leg. Age-matching naïve animals (*N* = 3) were used as a control of tumor development and progression **D,** Schematic of rechallenge. Right: tumor-free survival of rechallenged mice. **E,** individual tumor growth curves of rechallenged mice. **F,** Overall survival of the study. Blue background indicates the period postrechallenge. *, *P* < 0.05; ***, *P* < 0.001; ****, *P* < 0.0001; *ns*, nonsignificant.

The preliminary part of our work involved selecting a set of viruses from the 22 species included in the *Avulavirinae subfamily.* We selected isolates that comply with the biosafety requirements required for BSL-2 laboratory strains**,** have a confirmed complete genome sequence, lack pathogenicity in chickens and are able to be propagated in embryonated chicken eggs (31–37). Our final selection included prototype viruses from genus *Metaavulavirus* (APMV-2, -3, -6, -7, and -8)*, Orthoavulavirus* (APMV-9), *and Paraavulavirus* (APMV-3 and APMV-4; Fig. 1B; Table 1). The antitumor capacity of selected APMV viruses was tested *in vivo* in syngeneic B16-F10 melanoma (Fig. 2) and CT26.WT murine colon carcinoma tumor models (Fig. 3). Tumor-bearing mice received a total of four intratumoral injections (5 × 10^6^ PFU/50 μL PBS per dose) every other day. The oncolytic NDV LS-L289A virus was chosen as our reference for antitumoral activity and PBS mock-treated mice were used as our control group for tumor progression and survival.

In B16-F10 melanoma, all selected APMV viruses were able to restrain tumor growth during therapy (day 0 to day 6; Fig. 2B). Following treatment administration, APMV-7, APMV-8, and NDV LS-L289A treated-groups displayed comparable responses leading to a modest improvement on survival. APMV-4 treatment exhibited superior control over tumor growth, thus translating into the most significant benefit in extending survival (Fig. 2D).

In the CT26.WT colon carcinoma model, early suppression of tumor growth was observed in all the experimental groups leading to an overall benefit in survival when compared with the PBS control group (Fig. 3A and B). Sustained regressions that ultimately resolved into complete tumor elimination (CR, or complete response) were achieved by a subset of mice treated with APMV-2, -4, -6, -8, -9, and LS-L289A viruses (Fig. 3C). Considering that NDV virotherapy is known to elicit tumor-specific memory (38), we aimed to elucidate whether the complete tumor elimination fulfilled by other APMV viruses would entail long-lasting protection as well. To do so, tumor-free surviving animals were reimplanted with CT26.WT cells in the contralateral hind leg without further treatment on day 130 of the study. Naïve age-matching BALB/cJ mice were used as a control for tumor growth and survival (Fig. 3D). All control mice developed tumors and reached our experimental endpoint within 16 days postimplantation.

The only surviving animal of our APMV-2 group, as well as 1 of 3 mice from APMV-6 and -8 groups, failed to counteract the development of new tumors. Complete protection after rechallenge was observed in all long-term survivors fromNDV LS-L289A (*N* = 1), APMV-4 (*N* = 4), and APMV-9 (*N* = 3) groups. Remarkably, APMV-4 demonstrated the best control of tumor growth leading to complete tumor elimination and long-term antitumor protection in 80% of mice.

### Design and Development of a Recombinant APMV-4 Duck/Hong Kong/D3/1975 Virus by Reverse Genetics

**FIGURE 4:**
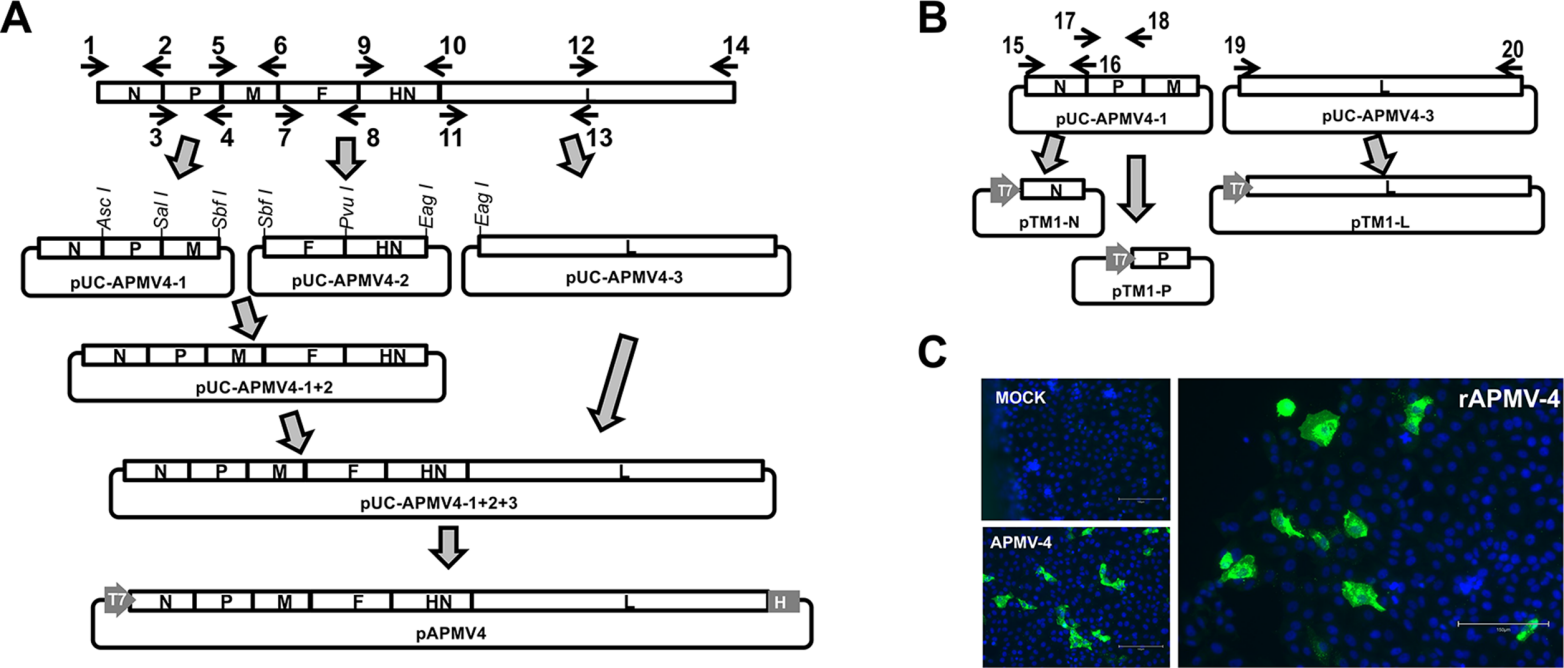
Rescue system strategy of the novel recombinant rAPMV-4 using anmplification and cloning strategy. Construction of the rescue plasmid pAPMV4 (A) and helper plasmids (B). Primer numbers correspond to the numbers shown in Table 1. **T7**: T7 RNA polymerase promoter sequence. **HdR:** hepatitis delta ribozyme. Size of the viral genes is shown to scale. **C,** Detection of the infectious clone rAPMV-4 on Vero cells by IF. Scale bar 150 μmol/L.

APMV-4 demonstrated superior inherent oncolytic capacity *in vivo* when compared with other selected isolates, including the clinical candidate NDV LS-L289A. This solid response positioned the APMV-4 Duck/Hong Kong/D3/1975 isolate as the strongest candidate for follow-up. Accordingly, we generated an infectious clone of APMV-4 by designing a plasmid-based rescue strategy modeled after the already established system for NDV and other paramyxoviruses (39). Briefly, a pAPMV4 rescue vector containing a full-length antigenomic cDNA of the Duck/Hong Kong/D3/1975 isolate was generated following a multistep cloning strategy using purified viral RNA as template (Fig. 4A). In parallel, the cloned cDNA was used to generate three helper vectors pTM-APMV4-N, pTM-APMV4-P, and pTM-APMV4-L, encoding the viral proteins N, P, and L, which conform the replication machinery of the virus (Fig. 4B). In both expression constructs, the cDNA is under the transcriptional control of a T7 promoter. To recover the infectious clone rAPMV-4, BSR-T7 cells preinfected with an attenuated MVA-T7 and then transfected with the rescue pAPMV4 and helper plasmids. A total of 24 hours after transfection, the cells and supernatants were inoculated into 8-day-old embryonated chicken eggs. Three days postinoculation, the allantoic fluid was harvested and the presence of infectious rAPMV4 was confirmed by immunofluorescence in cells infected with the harvested allantoic fluid (Fig. 4C).

### *In Vitro* Characterization of APMV-4 Oncolytic Features

**FIGURE 5:**
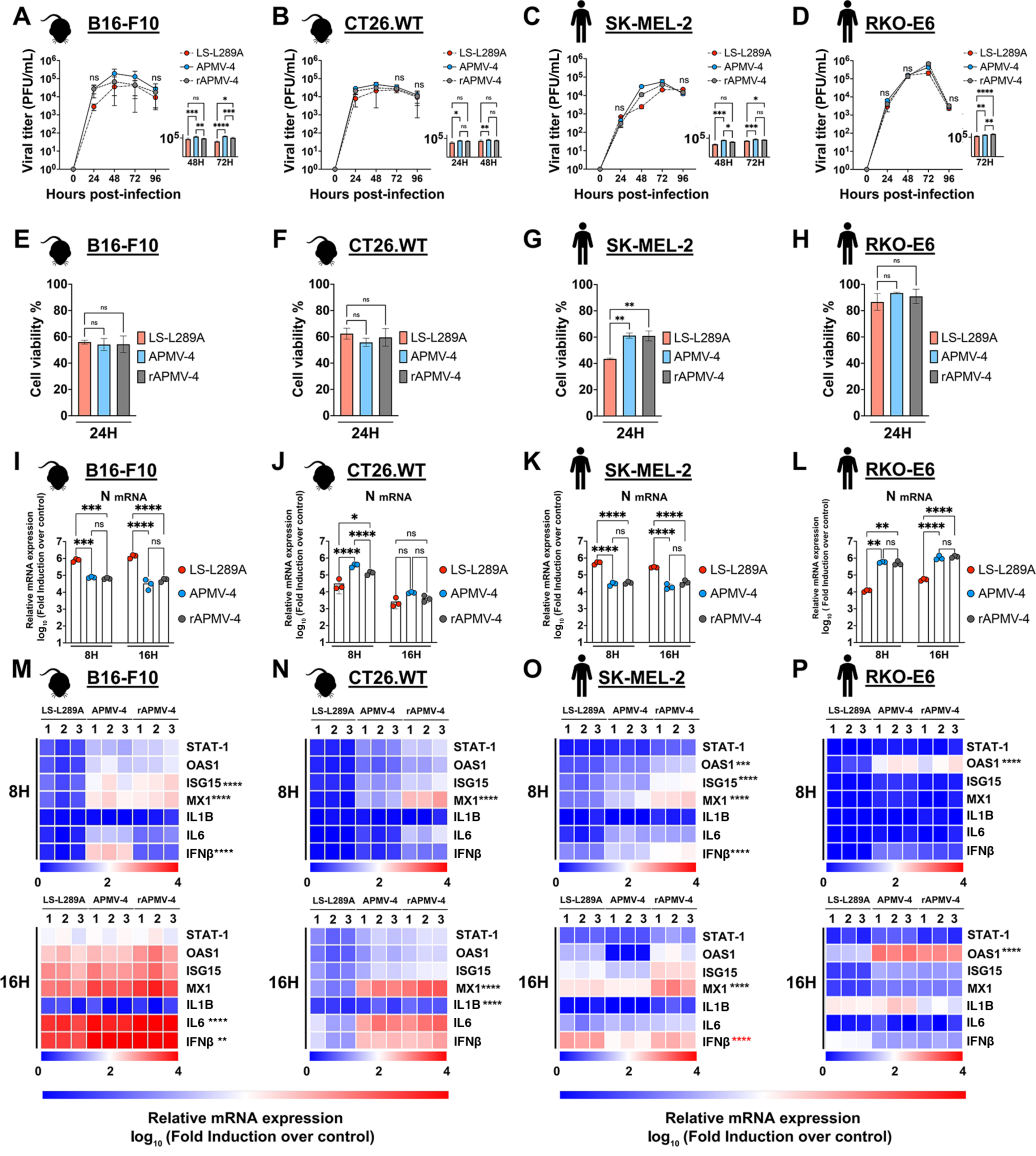
*In vitro* oncolytic activity of APMV-4 in murine and human cancer types. **A–D,** Replication analysis (growth curve kinetics). Cancer cells were cocultured with the indicated virus at a MOI of 0.1. Supernatants were tested for viral titers every 24 hours. Data represent the average titer obtained from three independent biological samples ± SD at the specified timepoint. **E–H,** Cytotoxicity. Cancer cells were infected with indicated virus or mock infected at a MOI of 1. MTT analysis of cell viability was performed 24 hours postinfection. Data represent the average of three independent biological samples ± SD. **I–P,** Transcription analysis of viral replication and proinflammatory genes by qPCR. Cancer cells were infected at a MOI of 1 or mock-infected and subjected to RNA extraction at 8- and 16-hours postinfection. **I**–**J,** Viral replication levels measured as mRNA expression of the N protein. Bars represent the average of three independent biological samples ± SD. **M–P**, Heatmaps showing levels of induction of IFNβ, ISGs (STAT1, ISG15, MX, OAS-1), and proinflammatory cytokines (IL6 and IL1B) for each independent biological samples (1, 2, 3) corresponding to **I**–**J** panels. Expression levels for each individual gene were calculated as log_10_ of fold induction over mock-infected cells. Two-way ANOVA analysis: *, *P* < 0.05; ***, *P* < 0.001; ****, *P* < 0.0001; *ns*, nonsignificant.

To assess the direct effect that APMV-4 has in tumor cells, we examined the replication capacity, cytotoxicity, and proinflammatory responses exerted by both WT and recombinant APMV-4 on melanoma and colon carcinoma cell lines of murine and human origin *in vitro* (Fig. 5). In these studies, mock-treated and NDV LS-L289A–treated cells were used as comparative references.

To study the replication capacity of APMV-4 and rAPMV-4 viruses, cancer cell monolayers were infected at a low MOI (0.1 PFU/cell) and maintained in postinfection media supplemented with TPCK-trypsin, necessary to allow for multicycle replication of lentogenic viruses *in vitro*. Every 24 hours, cell culture supernatants were tested for viral titers through immunofluorescence in Vero cells. APMV-4 and rAPMV-4 showed comparable growth kinetics and average peak titers in all the selected cancer cell lines (Fig. 5A–D). For both viruses, the highest viral titers were reached at 48 hours postinfection in the murine cancer cells lines (Fig. 5A and B) and at 72 hours in the human lines (Fig. 5C and D). Two-way ANOVA statistical analysis showed significant differences in replicative fitness between APMV-4 and LS-L289A viruses in a time-dependent and cell-specific manner (Fig. 5A–D). At those specific timepoints, AMPV-4 replication outperformed the replication of the recombinant LS-L289A virus.

Virus-mediated cytotoxicity and proinflammatory responses were assessed on cancer cell monolayers infected at a MOI of 1 PFU/cell and incubated for up to 24 hours in absence of TPCK-trypsin, thus limiting the viral replication to a single cycle. Analysis of cell viability by MTT assay at 24 hours postinfection showed no differences in cytolytic activity between APMV-4 and rAPMV-4. Both viruses were able to reduce the viability of B16-F10, CT26.WT, and SK-MEL-2 cell cultures to 60% and to induce 10%–15% viability loss in RKO-E6 cells (Fig. 5E–H). SK-MEL-2 cells showed higher susceptibility to LS-L289A virus, being the only cancer cell line in which significant differences in cytolytic activity were observed between the APMV-4s (40% viability loss) and the LS-L289A virus (60% loss; Fig. 5G).

The proinflammatory response elicited by APMV-4–infected cancers was evaluated at 8 and 16 hours postinfection (Fig. 5I–P). mRNA expression analysis by qPCR showed increased upregulation of INFβ, STAT-1, ISG15, OAS1, and MX1 genes by APMV-4–infected cells, when compared with the expression levels induced by LS-L289A at 8 hours post-nfection. This earlier and stronger type-I IFN signature was displayed by all cancer cell lines independently of their origin, and this signature was replicated by rAPMV-4 infection. At either 8 or 16 hours, significant differences between APMV-4 viruses and NDV were found in the expression of ISG-15 and MX-1. IL6 was particularly upregulated in murine cancer cells, while OAS1 was significantly upregulated by human cancer cells. Analysis of mRNA expression levels of the viral nucleoprotein N (Fig. 5I–L) did not show a direct association between the viral replication activity and the early immune signatures, with B16-F10 (Fig. 5I and M) and SK-MEL-2 melanoma cancer cells (Fig. 5K and O) showing higher levels of N mRNA of the LS-L289A virus, but a stronger immune stimulation in response to APMV-4 and rAPMV-4.

Finally, experimental infection of normal cells of human origin by LS-L289A, APMV-4, and rAPMV-4 (Supplementary Fig. S1) resulted into stronger upregulation of genes involved in antiviral response, as measure by the expression levels of IFNβ and MX-1 (Supplementary Fig. S1C–S1F). Thus, in combination with the low impact on cell viability (Supplementary Fig. S1A and S1B) is indicative that normal cells can more efficiently counteract the progression of viral infection.

### rAPMV-4 Preserves the Inherent Antitumor Capacity of the Parental APMV-4 Duck/Hong Kong/D3/1975 Virus

**FIGURE 6:**
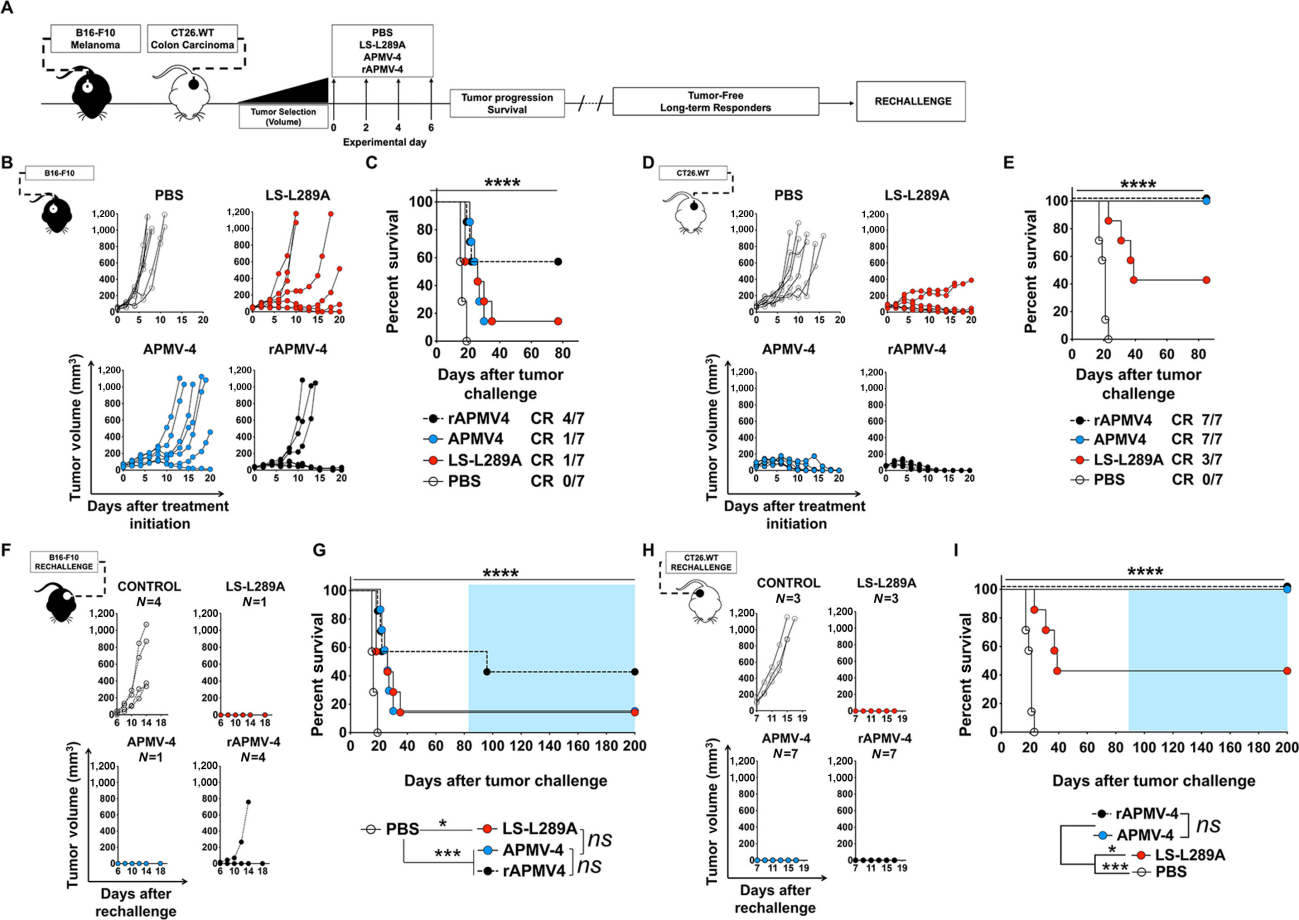
*In vivo* antitumor effect of novel rAPMV-4. **A,** Schematic representation of the study. B16-F10 melanoma and CT26.WT colon carcinoma tumor-bearing mice were intratumorally treated every other day with a total of four doses of 10^7^ PFU of LS-L289A, APMV-4, rAPMV-4 or PBS for control mice (days 0, 2, 4, and 6). Tumor volume was monitored every 48 hours. EEP of 1,000 mm^3^. **B** and **C,** Therapeutic response in B16-F10 murine melanoma model. **B,** Individual tumor growth curves. Each point represents tumor volume per mice at the indicated timepoint. **C,** Overall survival of treated B16-F10 tumor-bearing mice; CR, complete response. *P* value obtained through log-rank (Mantel–Cox) statistical analysis. *N* = 7 animals for each experimental group. **D** and **E,** Response in CT26.WT murine colon carcinoma tumor model. **D,** Individual tumor growth curves; each point represents tumor volume per mice at the indicated time point. **E,** Overall survival of treated CT26.WT tumor-bearing mice; CR, complete response. *P* value obtained through log-rank (Mantel–Cox) statistical analysis. *N* = 7 animals for each experimental group. **F**–**I,** Rechallenge. Tumor-free long-term responders were rechallenged in the contralateral hind leg with 5 × 10^5^ cancer cells. Age-matching naïve animals were used as a control of tumor development and progression. **F** and **G,** Rechallenge of B16-F10 long-term responders. **F,** Individual tumor growth curves. Age-matching control mice *N* = 4. **G,** Overall survival of B16-F10 study. Blue background indicates the period postrechallenge. **H–I,** Rechallenge of CT26.WT long-term responders. **H,** Individual tumor growth curves. **I,** Overall survival of CT26.WT study. Blue background indicates the period postrechallenge. *P* value obtained through log-rank (Mantel–Cox) statistical analysis. *, *P* < 0.05; ***, *P* < 0.001; ****, *P* < 0.0001; ns, nonsignificant.

The antitumor capacity of rAPMV-4 was tested *in vivo* in syngeneic B16-F10 melanoma and CT26.WT murine colon carcinoma tumor models (Fig. 6A). In these studies, the viral dose per injection was 10^7^ PFU/50 μL PBS. At the time of treatment initiation, the volume of B16-F10 melanoma tumors was 40 mm^3^ and CT26.WT tumors were 60 mm^3^ in size. Under these experimental conditions, rAPMV-4 therapy led to a 57% CR rate in mice bearing B16-F10 melanomas, exceeding those achieved by APMV-4 and LS-L289A viruses (14% CR; Fig. 6B and C). In syngeneic colon carcinoma, APMV-4 and rAPMV-4 virotherapies resulted in 100% elimination of CT26.WT tumors, outmatching the therapeutic effect of LS-L289A (40% CR; Fig. 6D and E). Homologous rechallenge of tumor-free survivors (Fig. 6F–I) resulted in total protection against colon carcinoma and melanoma, except for one long-term survivor in the B16-F10 rAPMV-4–treated group. (Fig. 6F and G).

## Discussion

The main aim of this work was to unveil members within the avian avulaviruses group with inherent antitumor capacity. Considering that the outcome of oncolytic virotherapy depends on the engagement of the host innate and adaptive immune systems (40), we opted for a straightforward *in vivo* antitumor capacity screening to investigate the therapeutic potential of various APMVs. In our studies, immunocompetent tumor-bearing mice were intratumorally inoculated with our selected isolates following a therapeutic strategy modeled after treatment regimens used by our group and others to study the efficacy of NDV (41–43). As our reference for anticancer activity, we employed the already characterized recombinant oncolytic NDV LaSota-L289A virus (24, 29). To identify APMVs with similar—or greater oncolytic capacity than the LS-L289A virus, we utilized all our viruses at a dose known to be suboptimal for NDV (5 × 10^6^ PFU/dose), where we have previously seen a moderate extension in survival in syngeneic B16-F10 tumors and up to 10% CRs in CT26.WT colon carcinoma. To account for the susceptibility and variability of responses to OV therapy in different cancers, our studies were performed in two distinct tumor models: B16-F10 melanoma and CT26.WT colon carcinoma (Figs. 2 and 3). Overall, the results of our *in vivo* screening highlight the potential of the *Avulavirinae* family as a source of antitumor virotherapeutics (Table 2). While all the selected APMVs demonstrated the ability to delay tumor growth during treatment administration (Fig. 2A and B; Fig 3A and B), the analysis of long-term survival and complete remissions underlined the contrasting antineoplastic phenotypes displayed by individual APMVs depending on the type of cancer (Fig. 2D and Fig. 3C). For example, the antitumor capabilities of the APMV-9 New York isolate—most closely related to NDV—elicited a response similar to PBS in melanoma but was capable of achieving 60% CR and protection against rechallenge in CT26.WT masses. Colon carcinoma tumors showed higher susceptibility to all APMVs (Fig. 3). This differential response to APMV treatment could be due to the contrasting genetic background of these cancers. CT26.WT cells are characterized by an oncogenic version of *Kras*, MEK (*Mapk1*) amplification and high expression levels *Nras* (44). Previous works have demonstrated that hyperactivation of Ras/Raf/MEK/ERK signaling sensitizes cells to oncolytic NDV (45–47). The susceptibility caused by this feature seems to extend to other APMVs, as indicated by the remarkable performance of multiple isolates in the colon carcinoma model. Among the viruses included in these studies, the APMV-4 isolate has shown the greatest antitumor capacity in both cancer models, exceeding the therapeutic potential of all other selected viruses, including the NDV LS-L289A. The advantage of APMV-4 over the LS-L289A virus was further validated in subsequent studies, where experimental conditions were adapted to favor the response of LS-L289A virus by increasing the viral dose to 10^7^ PFU (Fig. 6) and, additionally, where the initial tumor volume size was selected to be either more permissive (B16-F10 tumors; Fig. 6B–G) or more challenging (CT26.WT tumors; Fig. 6D–I) for the viruses to achieve complete therapeutic responses.

**TABLE 2:** Summary of therapeutic responses achieved by selected APMVs

	B16-F10 MELANOMA (Average survival control PBS = 13 days)				CT26.WT COLON CARCINOMA (Average survival control PBS = 20 days)			
	Median survival^a^	Survival vs. NDV^b^	% CR	% P.A.R	Median survival^a^	Survival vs. NDV^b^	% CR	% P.A.R
**APMV-2**	**15 days**	***ns***	**0%**	**N.A**	**26 days**	***ns***	**20%**	**N.A**
**APMV-3**	**16 days**	***ns***	**0%**	**N.A**	**24 days**	***ns***	**0%**	**N.A**
**APMV-6**	**17 days**	***ns***	**0%**	**N.A**	**144 days**	***ns***	**60%**	**66%**
**APMV-7**	**23 days**	***ns***	**0%**	**N.A**	**39 days**	***ns***	**0%**	**N.A**
**APMV-8**	**15 days**	***ns***	**0%**	**N.A**	**144 days**	***ns***	**60%**	**66%**
**APMV-9**	**15 days**	***P* *=* *0.01* ^a^**	**0%**	**N.A**	**300 days**	***ns***	**60%**	**100%**
**APMV-4**	**28 days^c^**	***P* *=* *0.03* ^a^**	**0%**	**N.A**	**300 days^c^**	***P* *=* *0.003* ^d^**	**80%**	**100%**
	**26 days^d^**	***ns***	**14%**	**100%**	**200 days^d^**	***P* *=* *0.02* ^a^**	**100%**	**100%**
**LS L289A**	**17 days^c^**	**N.A**	**0%**	**N.A**	**39 days^c^**	**N.A**	**20%**	**100%**
	**26 days^d^**	**N.A**	**14%**	**100%**	**39 days^d^**	**N.A**	**42%**	**100%**
**rAPMV-4**	**96 days^d^**	***ns***	**57%**	**75%**	**200 days^d^**	***P* *=* *0.02* ^a^**	**100%**	**100%**

**NOTE: % CR:** percentage of complete remission; %P.A.R: percentage of protection after rechallenge.

^a^Kaplan–Meier survival analysis for each treatment group.

^b^log-rank comparative analysis of survival with of each group versus NDV LS-L289A.

^c^Screening study.

^d^Follow-up *in vivo* study with rAPMV-4.

APMV-4 Duck/Hong Kong/D3/1975 was the first identified APMV-4 virus and is considered the prototype strain of the species *Avian paraavulavirus* (2, 48). This isolate has typically been recovered from wild waterfowl worldwide, and occasionally from domestic ducks, geese, and chickens, although no clinical signs of disease were ever reported in these infected animals (49, 50). This avirulent phenotype has been confirmed by experimental inoculations of birds (Table 2) and mammals (51). In our hands, intranasal administration of a high dose of APMV-4 (10^7^ PFU) did not compromise the health of inoculated mice (Supplementary Fig. S2). A complete genome sequence and molecular characterization of the Duck/Hong Kong/D3/1975 strain has been reported previously (32). However, little is known about the molecular biology involved in the virus–host cell interactions. APMV-4′s RBP HN protein has hemagglutinin and neuraminidase activities and is predicted to recognize sialic acids. Its F protein has a monobasic cleavage site (DIPQR↓F) that, although resembling those in avirulent lentogenic NDV strains, has been suggested to capacitate APMV-4 for multicycle replication in certain cell lines *in vitro*, despite not displaying a canonical furin cleavage site. Up till now, the molecular basis behind this observation remains unknown, and whether this phenomenon is limited to specific cell types or experimental conditions is still unclear. In our replication studies in cancer cells, we were only able to follow multicycle replication with the addition of exogenous TPCK-Trypsin to the infectious media. We did observe, however, that APMV-4 was able to reach higher titers than the LS-L289A virus while exhibiting similar growth kinetics (Fig. 5A–D). Considering all of the above, the distinct dependency of APMV4’s F protein on proteolytic activation by either endogenous or secretory proteases could support these differences in viral fitness and, to some extent, be advantageous for the oncolytic activity of the virus *in vivo*.

In addition, APMV-4 has demonstrated its ability to trigger proinflammatory and death responses in infected cancer cells (Fig. 5E–P). This oncolytic capability is known to lead to the initiation of a local inflammatory response in the tumor microenvironment necessary for the stimulation of systemic innate and tumor-specific immune responses by the host (38, 40). When compared with NDV, we found APMV-4 to be a more potent immune stimulator, leading to an earlier and more robust upregulation of type-I IFN responses. Interestingly, this effect was preserved among the different cancer cells tested (Fig. 5M–P) and is independent of the levels of viral replication (Fig. 5I–L). Because this distinctive proinflammatory response may be a critical driver of the enhanced therapeutic effect of APMV-4 *in vivo,* deciphering the molecular biology behind the immunostimulatory capacity of AMPV-4 will be a core aim of our future investigations. To that end, and to answer other questions regarding APMV-4-tumor interactions, we have set up a reverse genetics system for APMV-4 (Fig. 4). The recovered rAPMV-4 infectious clone has shown that it retains the biological behavior (Fig. 5) and inherent antitumor capacity of the natural isolate (Fig. 6).

In summary, our investigation into the antitumor capacity of APMVs has led us to the discovery of APMV-4 Duck/Hong Kong/D3/1975 OV, a novel cancer therapeutic that naturally displays greater antitumor potential than the clinical candidate NDV. From our results, and with technology that enables us to further design and develop improved recombinant therapeutics, the APMV-4 platform has positioned itself as a competitive candidate for translation into the clinic as an anticancer therapeutic for solid tumors. For that matter, rAPMV-4 has the potential to be modified (i) to express transgenes that could allow for the development of cancer-specific therapies, (ii) to express immunotherapeutic molecules, such a cytokines or chemokines, or (iii) to improve already established checkpoint blockade–based therapies. Furthermore, rAPMV-4 could be used to substitute standards of care like radiotherapy in (iv) *in situ* tumor vaccination approaches, as well as used in (v) combination with small-molecule immunostimulators.

## Supplementary Material

Supplementary Table S1 APMV-4 reverse genetics system primers

Supplementary Fig. S1 Antiviral response to APMV-4 and rAPMV-4 in normal cells.

Supplementary Table S2 RT-qPCR Primers' sequences

Supplementary Fig. S2 Experimental inoculation of APMVs in mice
