# Supplementary material for: Avian Paramyxovirus 4 Antitumor Activity Leads to Complete Remissions and Long-term Protective Memory in Preclinical Melanoma and Colon Carcinoma Models
Source: Cancer Res Commun. 2022 Jul 7;2(7):602–15. doi: 10.1158/2767-9764.CRC-22-0025 (PMC9351398; doi:10.1158/2767-9764.CRC-22-0025)
Supplement: Supplementary Table S1 — APMV-4 reverse genetics system primers [file crc-22-0025-s01.docx]

**Supplementary Table 1. APMV-4 reverse genetics system primers**

|  | Primer name | Sequence |
| --- | --- | --- |
| 1 | **APMV4-PCR1-1-F** | CCAAGCTTGCATGCCACGAAAAAGAAGAATAAAAGGCA |
| 2 | **APMV4-PCR1-1-R** | GGGCGCGCCACTGAGTCTT |
| 3 | **APMV4-PCR1-2-F** | CTCAGTGGCGCGCCCCA |
| 4 | **APMV4-PCR1-2-R** | GATGTCGACGGACGGTGTG |
| 5 | **APMV4-PCR1-3-F** | CCGTCCGTCGACATCCCT |
| 6 | **APMV4-PCR1-3-R** | CGGTACCCGGGGATCCATCACCTGCAGGATTACAT |
| 7 | **APMV4-PCR2-1-F** | CCAAGCTTGCATGCCTAATCCTGCAGGTGATGAATCTG |
| 8 | **APMV4-PCR2-1-R** | GTTCGATCGTTTTTAATTAAAAAGG |
| 9 | **APMV4-PCR2-2-F** | TAAAAACGATCGAACTGAGG |
| 10 | **APMV4-PCR2-2-R** | CGGTACCCGGGGATCATTTTACGGCCGCTCAGGG |
| 11 | **APMV4-PCR3-F** | CCAAGCTTGCATGCCGAGCGGCCGTAAAATTAACAC |
| 12 | **APMV4-125-F** | ACAATCTAGATAATCTTGATATCTACCAGCAGC |
| 13 | **APMV4-125-R** | GATTATCTAGATTGTCAGAACCCATAAAGAATTTGG |
| 14 | **APMV4-PCR3-R** | CGGTACCCGGGGATCAAGAAATAAAAGACATATTTTTTATTAAATATTAATACG |
| 15 | **InF-N-APMV4-F** | CACGATAATACCATGGCTGGTGTCTTCTCCCAGTATG |
| 16 | **InF-N-APMV4-R** | TTAGGCCTCTCGAGCCTGCAGCTACAGTTCAAAGTCGGGTTGATAGTC |
| 17 | **InF-P-APMV4-F** | CACGATAATACCATGGATTTTACTGACATTGATGCTGTCAACTC |
| 18 | **InF-P-APMV4-R** | TTAGGCCTCTCGAGCCTGCAGCTAGAGCCCAAGGGCTTGTC |
| 19 | **InF-L-APMV4-F** | CACGATAATACCATGTCCTGTCAAGAAGGAACCCTTCC |
| 20 | **InF-L-APMV4-R** | TTAGGCCTCTCGAGCCTGCAGCTAAAGTGAGAGGTAGCCCCAACC |
