# Supplementary material for: Avian Paramyxovirus 4 Antitumor Activity Leads to Complete Remissions and Long-term Protective Memory in Preclinical Melanoma and Colon Carcinoma Models
Source: Cancer Res Commun. 2022 Jul 7;2(7):602–15. doi: 10.1158/2767-9764.CRC-22-0025 (PMC9351398; doi:10.1158/2767-9764.CRC-22-0025)
Supplement: Supplementary Fig. S1 — Antiviral response to APMV-4 and rAPMV-4 in normal cells. [file crc-22-0025-s02.docx]

**Supplementary Figure 1**

**
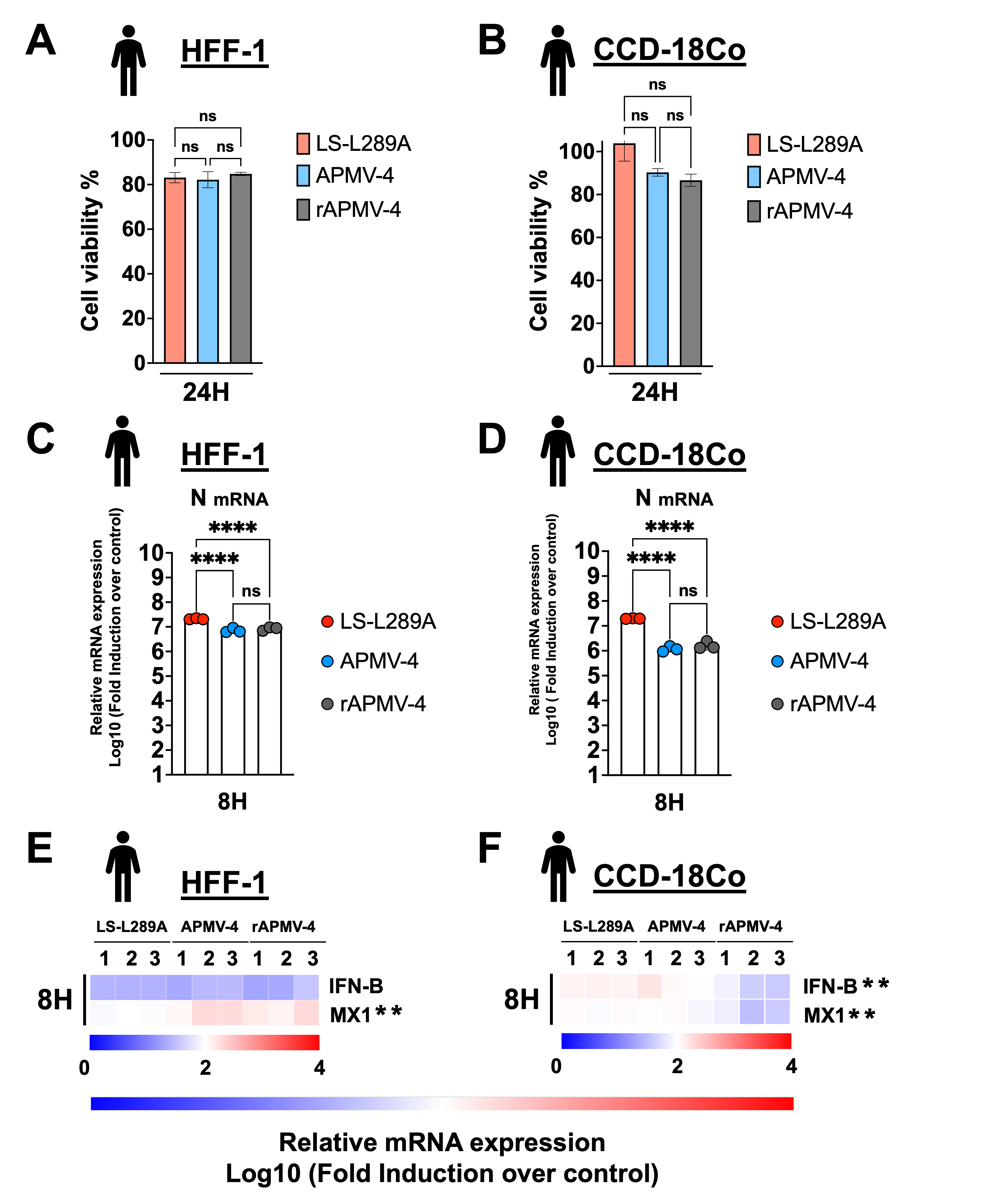
**

**Antiviral response to APMV-4 and rAPMV-4 in normal cells. A-B,** Cytotoxicity.
Human HFF-1 (skin fibroblast) and CDD-18Co (colon fibroblast) normal cells were infected with the indicated virus or mock-infected at a MOI of 1. MTT analysis of cell viability was performed 24 hours post-infection. Data represents the average of three independent biological samples ± SD. **C-F,** Transcription analysis of viral replication and antiviral-proinflammatory genes by qPCR**.** HFF-1 and CDD-18Co cells were infected at a MOI of 1 or mock-infected and subjected to RNA extraction at 8 hours post-infection. C-D**,** Viral replication levels measured as mRNA expression of the N protein. Bars represent the average of three independent biological samples ± SD. E-F, Heat maps showing levels of induction of IFN-β and MX1 genes for each independent biological samples (1, 2, 3). Expression levels were calculated as Log10 of Fold induction over mock infected cells. Two-way ANOVA analysis: **p* <0.05; ****p* <0.001; *****p* <0.0001; *ns*: non-significant.
