# Supplementary material for: Avian Paramyxovirus 4 Antitumor Activity Leads to Complete Remissions and Long-term Protective Memory in Preclinical Melanoma and Colon Carcinoma Models
Source: Cancer Res Commun. 2022 Jul 7;2(7):602–15. doi: 10.1158/2767-9764.CRC-22-0025 (PMC9351398; doi:10.1158/2767-9764.CRC-22-0025)
Supplement: Supplementary Table S2 — RT-qPCR Primers' sequences [file crc-22-0025-s03.docx]

**Supplementary Table 2. RT-qPCR Primers' sequences**

| Gen | Forward primer sequence | Reverse primer sequence | Species |
| --- | --- | --- | --- |
| IFN-β | CAGCTCCAAGAAAGGACGAAC | GGCAGTGTAACTCTTCTGCAT | Mouse |
| IFN-β | TCTGGCACAACAGGTAGTAGGC | GAGAAGCACAACAGGAGAGCAA | Human |
| IL-6 | CTGCAAGAGACTTCCATCCAG | AGTGGTATAGACAGGTCTGTTGG | Mouse |
| IL-6 | AGAGGCACTGGCAGAAAACAAC | AGGCAAGTCTCCTCATTGAATCC | Human |
| IL-1β | CTCGCCAGTGAAATGATGGCT | GTCGGAGATTCGTAGCTGGAT | Human |
| IL-1β | TGGGCTGGACTGTTTCTAATGC | TGTCTTGGCCGAGGACTAAGG | Mouse |
| ISG-15 | GGTGTCCGTGACTAACTCCAT | TGGAAAGGGTAAGACCGTCCT | Mouse |
| ISG-15 | TCCTGGTGAGGAATAACAAGGG | GTCAGCCAGAACAGGTCGTC | Human |
| MX1 | GACCATAGGGGTCTTGACCAA | AGACTTGCTCTTTCTGAAAAGCC | Mouse |
| MX1 | GTTTCCGAAGTGGACATCGCA | GAAGGGCAACTCCTGACAGT | Human |
| STAT-1 | TCACAGTGGTTCGAGCTTCAG | GCAAACGAGACATCATAGGCA | Mouse |
| STAT-1 | ATGTCTCAGTGGTACGAACTTCA | TGTGCCAGGTACTGTCTGATT | Human |
| N | GAACCATGTTGCCCTTGCAG | CCTCTCCAGGGTATCGGTGA | LS-L289A |
| N | ATCGGTCCTTAGCAGGAGGA | GGGTCCAGTCGTTGACACTT | APMV-4 |
