# Supplementary material for: Avian Paramyxovirus 4 Antitumor Activity Leads to Complete Remissions and Long-term Protective Memory in Preclinical Melanoma and Colon Carcinoma Models
Source: Cancer Res Commun. 2022 Jul 7;2(7):602–15. doi: 10.1158/2767-9764.CRC-22-0025 (PMC9351398; doi:10.1158/2767-9764.CRC-22-0025)
Supplement: Supplementary Fig. S2 — Experimental inoculation of APMVs in mice [file crc-22-0025-s04.docx]

**Supplementary Figure 2**

**Experimental inoculation of APMVs in mice.** C57BL/6J mice 6-8 weeks of age were anesthetized by intraperitoneal administration of a ketamine (90-100 mg/kg of body weight) and xylazine (10 mg/kg of body weight) sedation mixture. A suspension of 10^7^ PFU/50μl of each APMV isolate or PBS was intranasally administrated to anesthetized mice. Body weights were taken daily for 12 consecutive days. Graph: body weight loss/gain presented as percentage change from baseline (Day 0). Error bars in weight graph indicate mean ± SEM of five mice per group. Experimental end point for this study was set up at weight loss ≤ 20%.
